# Supplementary figures and images for: Contribution of the eye and of opn4xa function to circadian photoentrainment in the diurnal zebrafish
Source: PLoS Genet. 2024 Feb 26;20(2):e1011172. doi: 10.1371/journal.pgen.1011172 (PMC10919856; doi:10.1371/journal.pgen.1011172)

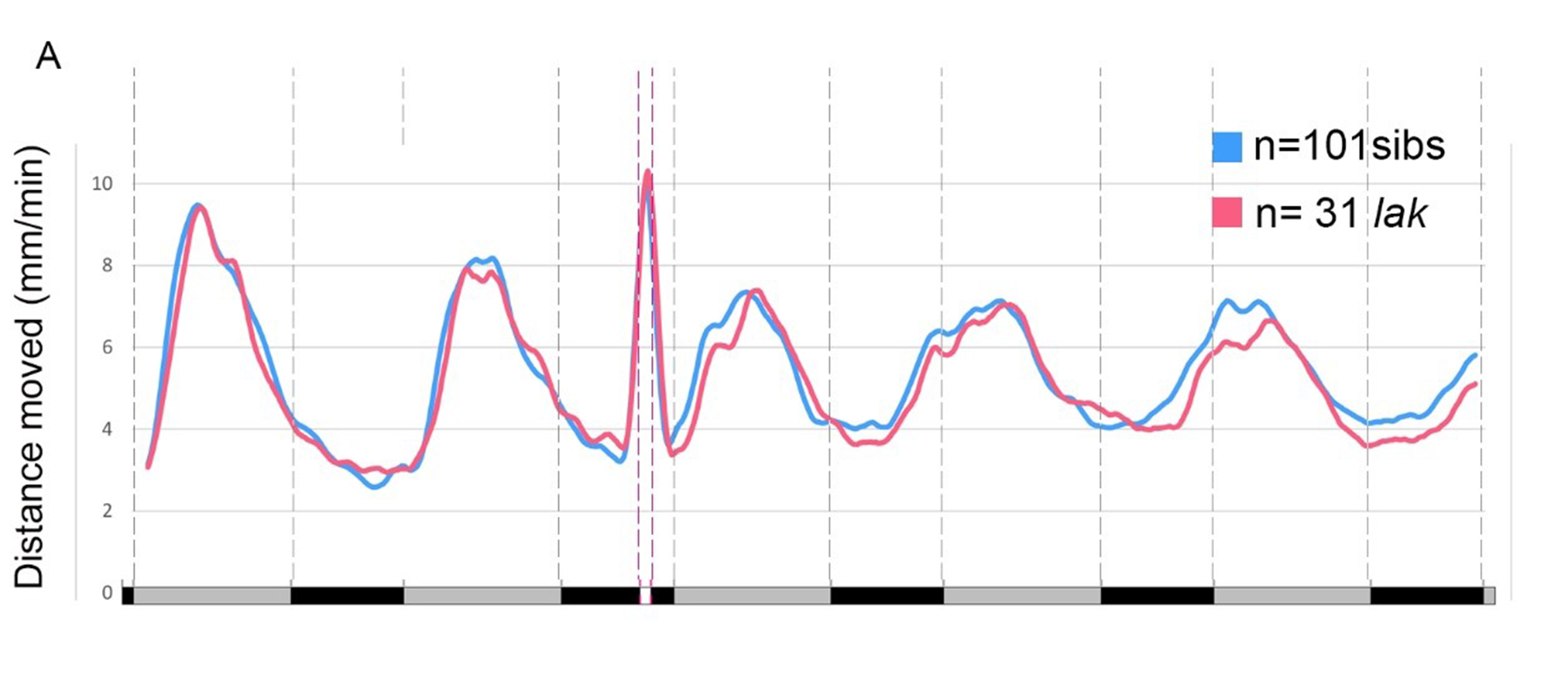

Supplement: S1 Fig — (A) Average distance moved merged from PA experiments in 10 min bins. Mean ± SE. The original data is the same than in Fig 2 but here, only the larvae for which a phase could be extracted for the two first and the two last cycles were included in the average. In addition, two rounds of smoothing each using ten successive time points were applied as this made the difference in phase shift between the lak and the sib larvae easier to visualize. (TIF) [file pgen.1011172.s001.tif]

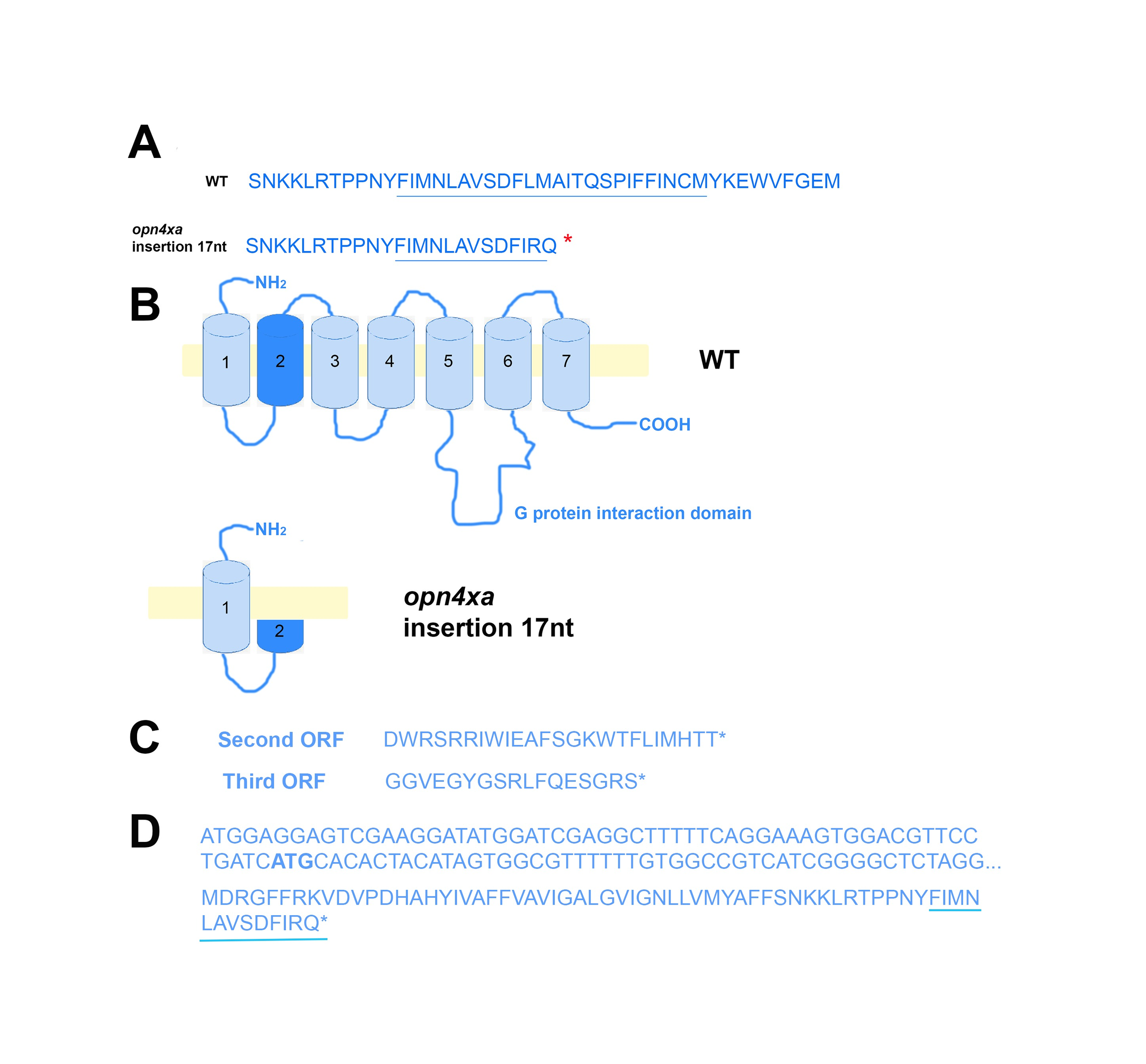

Supplement: S2 Fig — (A) Prediction of the protein sequences produced by the wt and mutant exon 2. The part corresponding to the second transmembrane domain (34) is underlined. The red asterisk indicates a premature stop codon. (B) Models of the WT and mutant predicted OPN4XA proteins. (C) Using alternative ORFs leads to a premature stop codon (indicated with a star). (D) The use of an alternative ATG (bold) also leads to a truncated protein. (TIF) [file pgen.1011172.s002.tif]

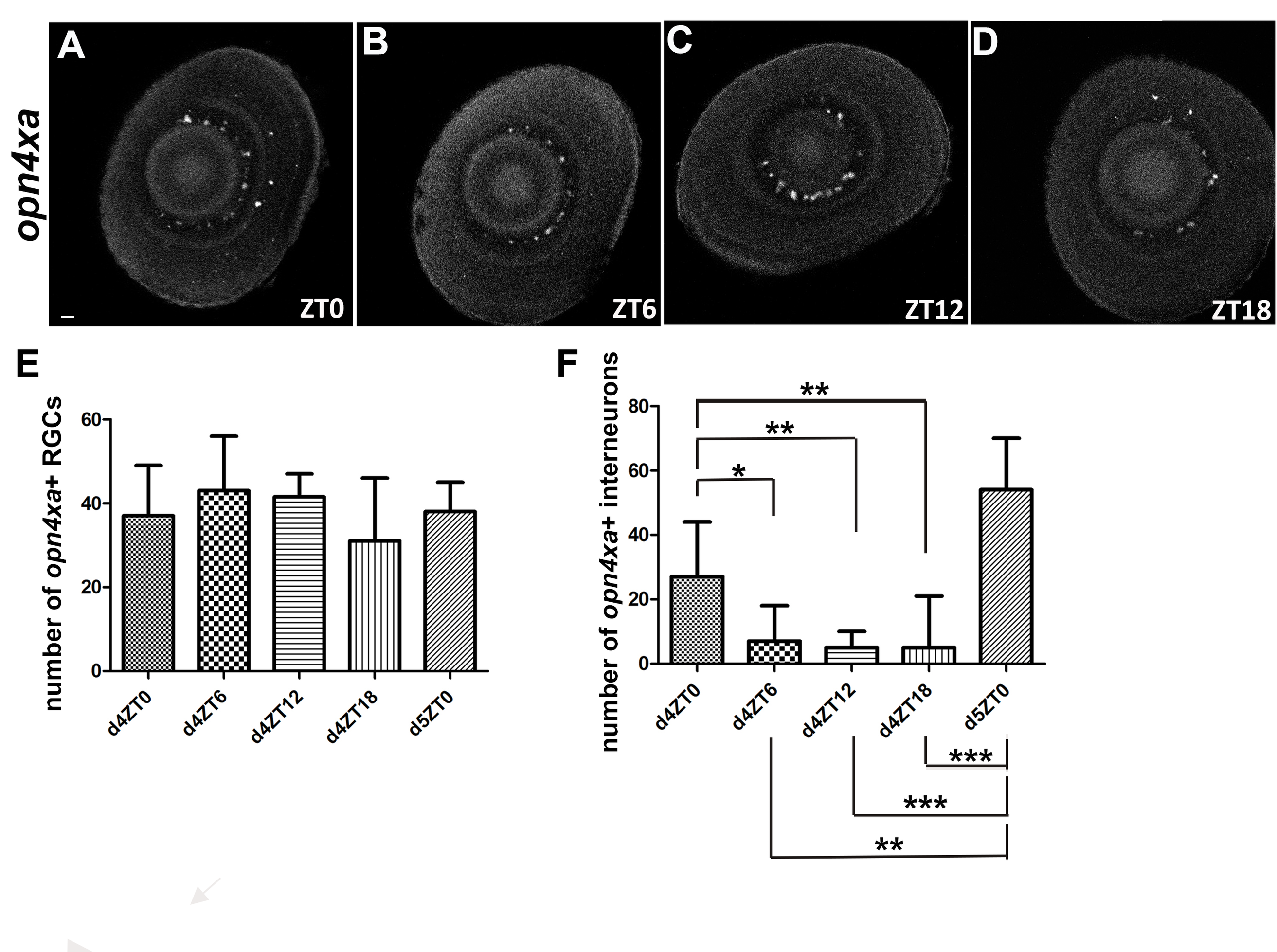

Supplement: S3 Fig — (A-D) Expression of opn4xa at 4days at different ZT using fluorescent in situ hybridization. Lateral view of mounted eyes imaged under the confocal microscope. The ventral side is oriented towards the left upper corner. Based on position, the opn4xa+ cells from the interneuron layer are most likely horizontal and amacrine cells. Scale bar: 10 μm. E) Number of opn4xa+ cells in the RGC layer in 96–128 hpf zebrafish larvae. All data follows a Gaussian distribution. No statistical differences were observed between the different time points using a one-way ANOVA with Bonferroni post hoc test. F) Number of opn4xa+ cells in the interneuron layer in 96–128 hpf zebrafish larvae. The data at 4dZT0 does not follow a Gaussian distribution. * p<0.05. ** p<0.001. *** p<0.0005 using a Kruskal-Wallis test with Dunn’s post hoc comparison. (TIF) [file pgen.1011172.s003.tif]

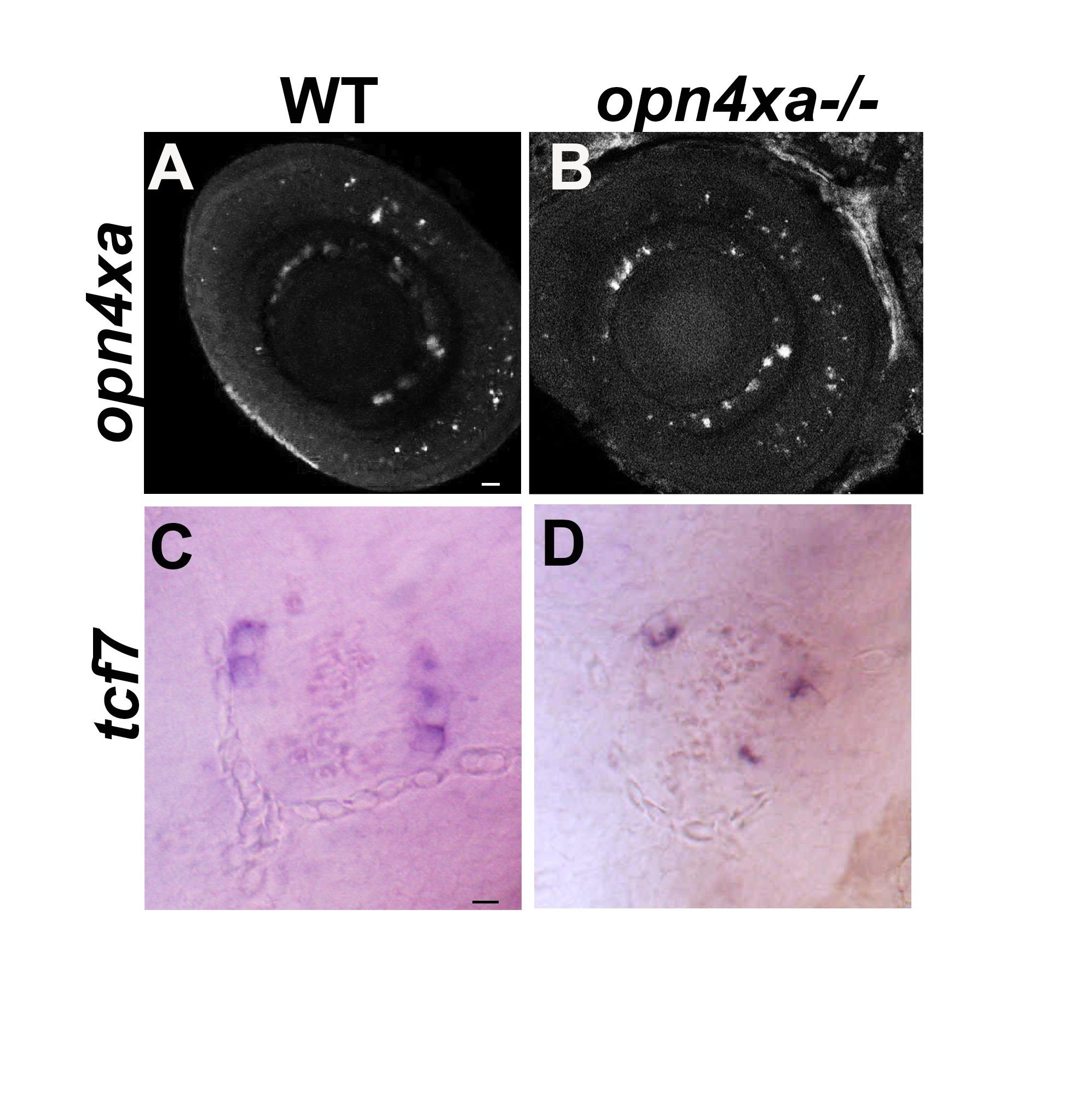

Supplement: S4 Fig — Characterization of opn4xa-/- retina and pineal glands. (A-B) Expression of opn4xa in the retina of wt and opn4xa-/- larvae at 4days ZT0. The ventral side is oriented towards the downward left corner. (C-D) Expression of tcf7 in the pineal gland at 6 days using in situ hybridization in wt and opn4xa-/- larvae. Dorsal views are shown. Anterior is up. Scale bar: 10 μm. (TIF) [file pgen.1011172.s004.tif]

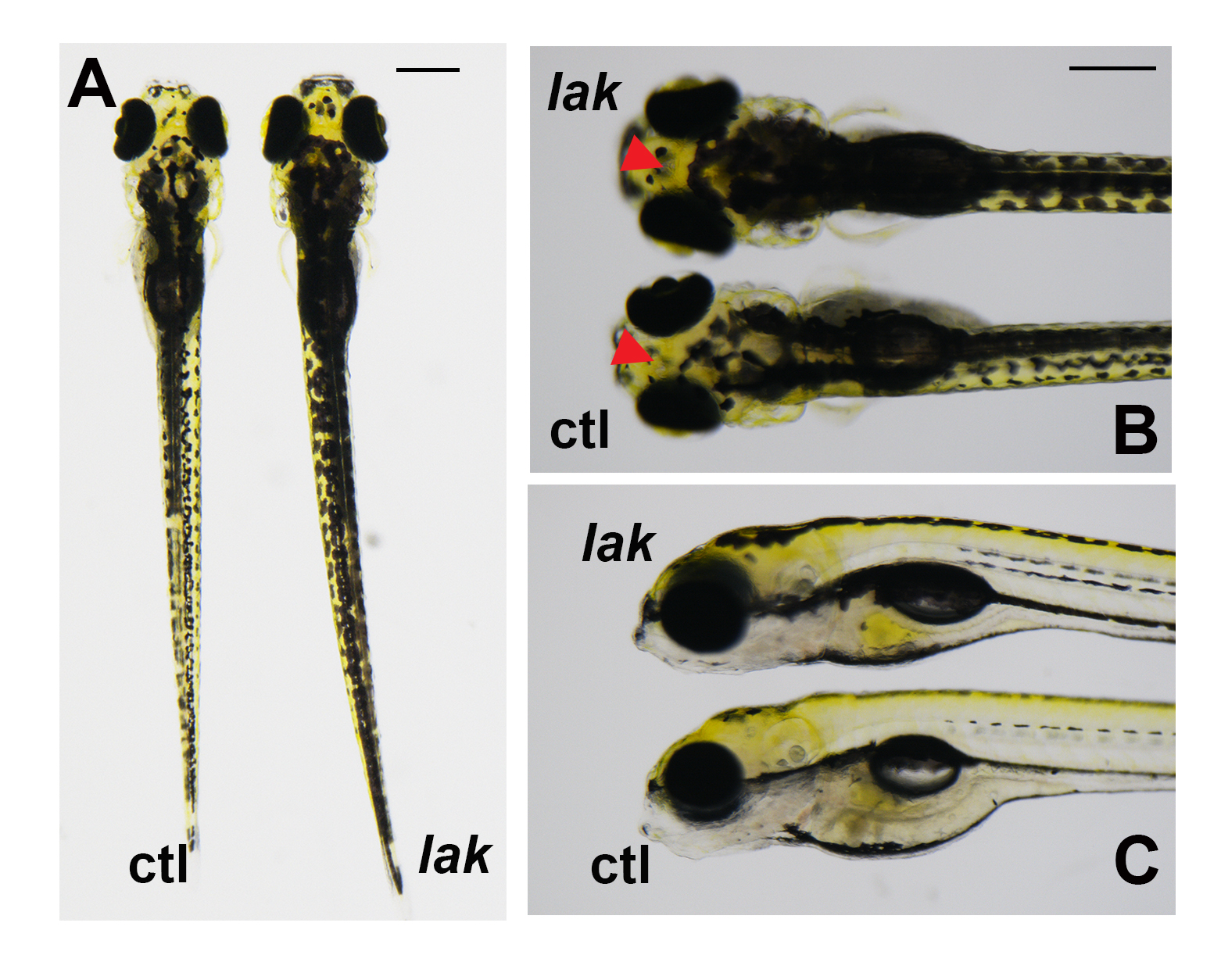

Supplement: S5 Fig — Live lak and sibling larvae at 5 dpf showing the differences in pigmentation. (A-B) Dorsal views. (C) side views. The red arrow in B points to the position of the pineal gland which is not covered by pigments in lak and sib larvae. Scale bar: 0.5 mm. (TIF) [file pgen.1011172.s005.tif]
