# Supplementary material for: Contribution of the eye and of opn4xa function to circadian photoentrainment in the diurnal zebrafish
Source: PLoS Genet. 2024 Feb 26;20(2):e1011172. doi: 10.1371/journal.pgen.1011172 (PMC10919856; doi:10.1371/journal.pgen.1011172)
Supplement: S1 Table — Activity of lakritz -/- versus control larvae in LD showing the average distance travelled (mm/min) over a 10 min window averaged during the day (D) or the night (N) periods. Mean ± S.D. D1 corresponds to the first day. The p value and statistical significance using a two-tailed Mann-Whitney test is indicated. (DOCX) [file pgen.1011172.s006.docx]

**Supplemental table 1: activity of *lakritz* -/- versus control larvae in LD**

| **condition** | **ctrl (n=55)** | ***lakritz* (n=55)** | **p value** |
| --- | --- | --- | --- |
| D1 | 13.95 ± 9.76 | 9.72 ± 8.73 | **** 0.008** |
| N1 | 2.92 $\pm$ 2.28 | 2.85 ± 2.57 | n.s 0.42 |
| D2 | 16.90 ± 10.25 | 11.18 ± 7.40 | ***** 0.0005** |
| N2 | 4.04 ± 2.22 | 4.98 ± 4.22 | n.s 0.51 |
| D3 | 13.19 ± 8.56 | 10.88 ± 7.07 | n.s 0.13 |
| N3 | 3.56 ± 2.17 | 3.95 ± 2.94 | n.s 0.57 |
