# Supplementary material for: Contribution of the eye and of opn4xa function to circadian photoentrainment in the diurnal zebrafish
Source: PLoS Genet. 2024 Feb 26;20(2):e1011172. doi: 10.1371/journal.pgen.1011172 (PMC10919856; doi:10.1371/journal.pgen.1011172)
Supplement: S2 Table — The difference between the ctl DD and the lak DD populations is not significant using a khi-two test p = 0.957, similar results are obtained between the ctl LL and the lak LL, p = 0,91. (DOCX) [file pgen.1011172.s007.docx]

**Supplemental table 2: Percentage of rhythmic animals in *lak* -/- versus ctl animals**

| condition | % of rhythmic animals |
| --- | --- |
| Ctl DD (n=84) | 96,4% |
| *lak* DD (n=84) | 97,5% |
| Ctl LL (n=81) | 96,3% |
| *lak* LL (n=81) | 98,8% |
