# Supplementary material for: Contribution of the eye and of opn4xa function to circadian photoentrainment in the diurnal zebrafish
Source: PLoS Genet. 2024 Feb 26;20(2):e1011172. doi: 10.1371/journal.pgen.1011172 (PMC10919856; doi:10.1371/journal.pgen.1011172)
Supplement: S3 Table — Activity of lakritz -/- versus control larvae in DD showing the average distance travelled (mm/min) over a 10 min window averaged during the day (D) or the night (N) periods. Mean ± S.D. D1 corresponds to the first day. The p value and statistical significance using a two-tailed Mann-Whitney test is indicated. (DOCX) [file pgen.1011172.s008.docx]

**Supplemental table 3: activity of *lakritz* -/- versus control larvae in DD**

| **condition** | **ctrl (n=84)** | ***lakritz* (n=84)** | **p value** |
| --- | --- | --- | --- |
| D1 | 8.9 ± 8.7 | 8.73 ± 7.08 | n.s 0.43 |
| N1 | 2.78± 3.4 | 2.45 ± 1.67 | n.s 0.98 |
| D2 | 6.20 ± 4.18 | 6.39 ± 5.77 | n.s 0.44 |
| N2 | 2.64 ± 1.60 | 2.80 ± 5.64 | n.s 0.92 |
| D3 | 6.76 ± 7.25 | 6.86 ± 5.64 | n.s 0.95 |
| N3 | 3.13 ± 1.95 | 3.27± 2.16 | n.s 0.87 |
| D4 | 8.68 ± 8.41 | 8.52 ± 6.89 | n.s 0.55 |
| N4 | 3.42 ± 2.20 | 3.43 ± 2.18 | n.s 0.92 |
