# Supplementary material for: Contribution of the eye and of opn4xa function to circadian photoentrainment in the diurnal zebrafish
Source: PLoS Genet. 2024 Feb 26;20(2):e1011172. doi: 10.1371/journal.pgen.1011172 (PMC10919856; doi:10.1371/journal.pgen.1011172)
Supplement: S4 Table — Activity of lakritz -/- versus control larvae in LL showing the average distance travelled (mm/min) over a 10 min window averaged during the day (D) or the night (N) periods. Mean ± S.D. D1 corresponds to the first day. The p value and statistical significance using a two-tailed Mann-Whitney test is indicated. (DOCX) [file pgen.1011172.s009.docx]

**Supplemental table 4: activity of *lakritz* -/- versus control larvae in LL**

| **condition** | **ctrl (n=72)** | ***lakritz* (n=72)** | **p value** |
| --- | --- | --- | --- |
| D1 | 21.31±13.33 | 19.49±12.39 | n.s 0.44 |
| N1 | 7.38±4.59 | 6.61±4.83 | n.s 0.27 |
| D2 | 22.99±14.01 | 22.88±19.03 | n.s 0.27 |
| N2 | 8.03±4.35 | 9.28±6.59 | n.s 0.35 |
| D3 | 17.71±8.51 | 17.43±14.59 | n.s 0.096 |
| N3 | 8.11±3.63 | 8.54±5.38 | n.s 0.85 |
