# Supplementary material for: Contribution of the eye and of opn4xa function to circadian photoentrainment in the diurnal zebrafish
Source: PLoS Genet. 2024 Feb 26;20(2):e1011172. doi: 10.1371/journal.pgen.1011172 (PMC10919856; doi:10.1371/journal.pgen.1011172)
Supplement: S5 Table — Activity of opn4xa -/- versus control larvae in LD showing the average distance travelled (mm/min) over a 10 min window averaged during the day (D) or the night (N) periods. Mean ± S.D. D1 corresponds to the first day. The p value and statistical significance using a two-tailed Mann-Whitney test is indicated. (DOCX) [file pgen.1011172.s010.docx]

**Supplemental table 5: activity of *opn4xa* -/- versus control larvae in LD**

| **condition** | **wt (n=48)** | ***opn4xa-/-* (n=48)** | **p value** |
| --- | --- | --- | --- |
| D1 | 22.16 ± 10.02 | 22.42 ± 13.12 | n.s 0.73 |
| N1 | 9.41 ± 6.45 | 7.94 ± 4.99 | n.s 0.30 |
| D2 | 20.46 ± 7.96 | 19.49 ± 8.25 | n.s 0.50 |
| N2 | 7.27 ± 4.86 | 6.32 ± 4.53 | n.s 0.27 |
| D3 | 14.35 ± 5.19 | 12.52 ± 6.12 | n.s 0.07 |
| N3 | 5.59 ± 3.41 | 4.19 ± 3.09 | *** 0.01** |
