# Supplementary material for: Contribution of the eye and of opn4xa function to circadian photoentrainment in the diurnal zebrafish
Source: PLoS Genet. 2024 Feb 26;20(2):e1011172. doi: 10.1371/journal.pgen.1011172 (PMC10919856; doi:10.1371/journal.pgen.1011172)
Supplement: S6 Table — The difference between the WT DD and the opn4xa-/- DD populations is not significant using a khi-two test, p = 0.06. (DOCX) [file pgen.1011172.s011.docx]

**Supplemental table 6: percentage of rhythmic animals in *opn4xa*-/- versus WT animals**

| condition | % of rhythmic animals |
| --- | --- |
| WT DD (n=69) | 98,5% |
| *opn4xa-/-* DD (n=69) | 92,8% |
| WT LL (n=66) | 98,5% |
| *opn4xa -/-*LL (n=66) | 98,5% |
