# Supplementary material for: Contribution of the eye and of opn4xa function to circadian photoentrainment in the diurnal zebrafish
Source: PLoS Genet. 2024 Feb 26;20(2):e1011172. doi: 10.1371/journal.pgen.1011172 (PMC10919856; doi:10.1371/journal.pgen.1011172)
Supplement: S7 Table — Activity of opn4xa -/- versus control larvae in DD showing the average distance travelled (mm/min) over a 10 min window averaged during the day (D) or the night (N) periods. Mean ± S.D. D1 corresponds to the first day. The p value and statistical significance using a two-tailed Mann-Whitney test is indicated. (DOCX) [file pgen.1011172.s012.docx]

**Supplemental table 7: activity of *opn4xa* -/- versus control larvae in DD**

| **condition** | **wt (n=65)** | ***opn4xa-/-* (n=65)** | **p value** |
| --- | --- | --- | --- |
| D1 | 14.73 ± 7.04 | 16.26 ± 10.22 | n.s 0.41 |
| N1 | 6.54 ± 1.96 | 6.15 ± 2.27 | n.s 0.35 |
| D2 | 12.52 ± 5.10 | 13.23 ± 6.58 | n.s 0.32 |
| N2 | 5.62 ± 2.06 | 6.04 ± 2.59 | n.s 0.38 |
| D3 | 8.94 ± 2.24 | 9.09 ± 2.93 | n.s 0.58 |
| N3 | 5.49 ± 2.09 | 5.45 ± 2.21 | n.s 0.63 |
| D4 | 7.15 ± 2.35 | 7.34 ± 2.33 | n.s 0.69 |
| N4 | 5.13 ± 2.43 | 5.30 ± 2.01 | n.s 0.65 |
