# Supplementary material for: Contribution of the eye and of opn4xa function to circadian photoentrainment in the diurnal zebrafish
Source: PLoS Genet. 2024 Feb 26;20(2):e1011172. doi: 10.1371/journal.pgen.1011172 (PMC10919856; doi:10.1371/journal.pgen.1011172)
Supplement: S8 Table — Activity of opn4xa -/- versus control larvae in LL showing the average distance travelled (mm/min) over a 10 min window averaged during the day (D) or the night (N) periods. Mean ± S.D. D1 corresponds to the first day. The p value and statistical significance using a two-tailed Mann-Whitney test is indicated. (DOCX) [file pgen.1011172.s013.docx]

**Supplemental table 8: activity of *opn4xa* -/- versus control larvae in LL**

| **condition** | **wt (n=66)** | ***opn4xa-/-* (n=66)** | **p value** |
| --- | --- | --- | --- |
| D1 | 16.56 ± 9.57 | 20.15 ± 12.25 | n.s 0.07 |
| N1 | 3.84 ± 2.99 | 6.22 ± 5.60 | *** 0.02** |
| D2 | 16.67 ± 7.79 | 19.22 ± 11.49 | n.s 0.26 |
| N2 | 4.90 ± 3.30 | 5.92 ± 4.48 | n.s 0.36 |
| D3 | 14.27 ± 7.16 | 13.94 ± 7.26 | n.s 0.82 |
| N3 | 5.92 ± 4.09 | 5.63 ± 3.75 | n.s 0.70 |
