# Supplementary material for: Contribution of the eye and of opn4xa function to circadian photoentrainment in the diurnal zebrafish
Source: PLoS Genet. 2024 Feb 26;20(2):e1011172. doi: 10.1371/journal.pgen.1011172 (PMC10919856; doi:10.1371/journal.pgen.1011172)
Supplement: S9 Table — The right column indicates the ZDB gene ID (https://zfin.org). (DOCX) [file pgen.1011172.s014.docx]

**Supplemental table 9: qRTPCR primer sequences**

| **Gene** | **Forward primer** | **Reverse primer** | **Gene ID** |
| --- | --- | --- | --- |
| *bmal1a* | 5’TGTAACGCAATGTCTCGCAAG3’ | 5’GTGGCCCCTCGTAATGTCTTC3’ | 000509-1 |
| cry1a | 5’AGGCGTGGAGGTGATAGTTC3’ | 5’TGCTGA’TGAGGGTCTGGAAG3’ | 010426-2 |
| *dec1* | 5’CGCTGGATATCTCTGACATGCAAGG3’ | 5’CGTGAGTTTAAGGTGTTCGGGCAG3’ | 030131-3133 |
| *tefa* | 5’AACCCCTCCACCGAATCTTC3’ | 5’TCCCATTTCACTGACCCCTC3’ | 990415-264 |
| *per1a* | 5’GAGACGACGGAAAGCAAGAG3’ | 5’GCTCTGAACTTCCGCTCAAA3’ | 011220-1 |
| *per1b* | 5’ATGTGCAGGCTGTAGATCCC3’ | 5’CCGTCAGTTTCGCTTTTCTC3’ | 040419-1 |
| *nr1d2a* | 5’AGGACGCCATCAGTGTGTTT 3’ | 5’CTGGCCCAGGAAAACAAGTA3’ | 040504-1 |
| *per2* | 5’GCTTCACCACACCATACAGG 3’ | 5’GTCTGACGGGGACGAGTCT3’ | 011220-2 |
| *B-actin* | 5’GCCTGACGGACAGGTCAT 3’ | 5’ ACCGCAAGATTCCATACCC 3’ | 000329-1 |
