# Supplementary material for: Contribution of the eye and of opn4xa function to circadian photoentrainment in the diurnal zebrafish
Source: PLoS Genet. 2024 Feb 26;20(2):e1011172. doi: 10.1371/journal.pgen.1011172 (PMC10919856; doi:10.1371/journal.pgen.1011172)
Supplement: Attachment — Submitted filename: answer to reviewers.docx [file pgen.1011172.s026.docx]

Dear Dr Ewer and reviewers

We are submitting a revised version of our manuscript. Overall, we were happy that the reviewers found both the question and findings interesting and provided a list of improvements. Please find enclosed a point by point answer to the reviewers’ comments.

Please bear in mind that the line numbers correspond to the manuscript with highlighted changes and not the ‘text only’ version.

**Reviewer #1:**

Please bear in mind that the line numbers correspond to the manuscript with highlighted changes and not the ‘text only’ version.

*1. >As a general layout comment- it would help to include line and page numbers, because it makes it easier to refer to specific sentences in the manuscript.*

This was done, indeed it helps!

2. „*The eye in general and melanopsin expressing cells in particular are crucial for circadian rhythms in nocturnal mammals, most notably during photoentrainment, by which circadian rhythms adapt to a changing light environment.“*

*> Remove nocturnal. This is probably a general feature for mammals, irrespective of nocturnality/diurnality.*

Concerning the eye we have added a sentence in the introduction to report its role in human : ‘Similarly, in human, fifty percent of blind people exhibit circadian misalignement with the LD cycles (Hartley et al., 2018; Lockley et al., 2007; Sack et al., 1992)’.

Concerning ipRGCs and melanopsin, we agree with the reviewer that experiments performed in mice have led to the idea they are crucial for circadian control in mammals in general, based on the supposed conservation of neural circuits between different mammal species.

As requested, we have modified our summary: l 48-50 **: “**Experiments performed in mice have established a crucial role for the eye in general and melanopsin expressing cells in particular in the control of circadian rhythms most notably during photoentrainment, by which circadian rhythms adapt to a changing light environment”.

3. „*raising the intriguing possibility that vertebrates might employ different molecular/cellular circuits for photoentrainment depending on their phylogeny and/or temporal niche.“
> It is already well established that in zebrafish different peripheral cell types are directly photosensory. See work by the labs of David Whitmore, Nick Foulkes etc.. The authors even refer to the relevant literature in their introduction.
So, this is not raising a possibility, but it is a long-standing fact. The question is more to the mechanistic details, how different photoreceptors contribute to it, and why this ability apparently got lost in mammals. This is where the authors actually make their contributions. They might be not so obvious, but they are nevertheless important for the large picture of non-visual photoreceptor functions. So, it would be appropriate to re-word this summary accordingly.*
As mentioned by the reviewer and as detailed in the introduction, zebrafish peripheral cells are photosensitive and this sensitivity contributes to the entrainment of the molecular clock as shown by the Whitmore and Foulkes lab. The biological role of this peripheral photosensitivity is however unclear. For instance, it is possible that this peripheral photosensitivity, does not impact the photoentrainment of behavior but serves local functions. On the other hand, one could imagine that a behavior would require some level of central control and would be difficult to pilot from the periphery.

The sentence that the reviewer refers to specifically deals with the behavioral part of the circadian rhythms and not the molecular clock, which might not have been clear enough in our manuscript, as our point is to address the role of the eye and of the *opn4xa* photosensitivity of the pineal regarding photoentrainment of locomotor activity**.** We however agree that the sentence we used is too strong and tunned it down (l.54-56) to say that it **supports** rather than **raise** that “vertebrates might employ different molecular/circuits for photoentrainment of behavior depending on their phylogeny and/or temporal niche”.

*4.Introduction:*
*"Once established, these rhythms persist in constant light conditions, " > constant conditions, not just “light“ or better re-phase to: “conditions that don’t provide entrainment information, e.g. constant light and temperature.”*

l.63. The text has been modified for ‘constant conditions’.

*5."Although ipRGCs mediate circadian and direct effects of light on behaviour in nocturnal mammals, it is unclear if these roles are conserved in diurnal and non-mammalian species"*
*>The statements after these sentences need serious rewording. It is long established especially by the work of David Whitmore, Nicholas Foulkes and their labs that individual cells in fish are themselves light sensitive, independent of the eyes. The authors refer to Cavallari et al.. 2011, but do not embed the findings and conclusions of this paper well into their introduction. Directly after the citation they write: “While the function of the eye regarding circadian rhythms has not been addressed in fishes…” and “The role of this local photodetection regarding circadian behaviours has not been explored… “.
However, Cavallari et al.. 2011 explicitly states (in their summary!): “We reveal that eye loss does not account for this “blind” clock. Specifically, mutations of two widely expressed non-visual opsin photoreceptors (Melanopsin and TMT opsin) are responsible for the blind clock phenotype in the cavefish.”*

*Cavallari et al.. 2011 provides evidence for this statement by tissue culture experiments. Interestingly, Cavallari et al.. 2011 have not performed the lak- mutant experiments, done in this manuscript. So, the current study certainly adds information, but the precise value of these new experiments needs to be better worked out. Thus, the current introduction needs serious rewording.*

Indeed, Cavallari et al., 2011 have shown that the blind cavefish Phreatichthys andruzzii bears mutations in *opn4m2* and *tmt* opsin,which are predicted to produce non- functional proteins in this species and that reexpression of the non-mutated (zebrafish) forms in a cell line they derived from cavefish fin clips rescues the rhythmic light-induced expression of a *zfper2: luciferase* reporter (Fig.7 and 8). What **they did not show** is that re-expression of the non-mutated forms of these opsins in the animal rescues photoentrainment of their locomotor activity. Thus, when in their summary they refer to the “blind clock”, in our opinion, they are talking about the molecular clock of fin cells. While these findings are extremely important, it is important to note that, in contrast to the Cavallari analysis, we focuss on the control of behavior and locomotor activity, we have tried to make this issue clearer for the reader and modified the text accordingly: l.111 to 133:

“While the zebrafish has emerged as a powerful non-mammalian diurnal vertebrate model for chronobiology, the neural circuit controlling photoentrainment of behaviours has not been identified in this species. The observation that two species of cavefishes bearing eye degeneration *: Astyanax mexicanus* and Phreatichthys andruzzii do not show robust light-entrainable circadian locomotor activity rhythms could suggest that eye function is crucial for photoentrainment of locomotor rhythms in fishes (Cavallari et al., 2011). However, the observed circadian locomotor phenotypes could result from additional photoreceptive deficits. Indeed, Phreatichthys andruzzii present mutations in several opsin genes that are expressed in extra-ocular locations (Cavallari et al.. 2011). While the function of the eye regarding circadian rhythms of behaviours has not been addressed in fishes, in the zebrafish model, an additional level of complexity arises from the observation that all cell types are photosensitive (Carr and Whitmore, 2005; Vallone et al., 2004; Whitmore et al., 2000) . This local photodetection could serve local functions (metabolism, transcription, cell cycle) or participate to the control of behaviors, although some level of central control is expected for orchestrating a complex process such as behavior. As such, the relative importance of central versus peripheral control for the photoentrainment of locomotor activity is still unclear”.

*6."Our results suggest that the function of the retina and/or the intrinsic photosensitivity of ipRGCs-like-cells of the pineal gland " > Suggest to re-word to „ Our results suggest that the function of the retina and the intrinsic photosensitivity of ipRGCs-like-cells of the pineal gland expressing opn4xa …”.Alternatively- refer to the fos stainings and make clear that your data suggest that at these early stages opn4xa appears to be the only opsin mediating light input into the pineal with light of the given duration, specific spectrum and intensity.*

*There are more opsins expressed in the pineal gland, and hence without further specifications it is difficult to conclude from the lack of one of the opsins about the functionality of the entire cell type.*

Our summary (l.26-28**)** and introduction (l144-153) were rewritten as to better describe our previous work on the pineal gland that has shown that light induces two different responses in the pineal gland:

-an ON response that is elicited by blue light and white light in *opn4xa*+ projection neurons

-an OFF response that occurs in *opn4xa*- projection neurons and that is elicited either by white, blue, green and red light and that most likely requires the classical photoreceptors of the pineal signalling onto these projection neurons (Cau et al., 2019; Sapède et al., 2020). The ON response is impaired in the *opn4xa* mutant suggesting that it is controlled by *opn4xa* but we do not consider to have abolished the function of the pineal in this mutant as *opn4xa-* cells are implicated in an OFF response.

*7.Results:*
*>Please add information on the age of the tested fish into Fig1. It’s in the methods, but as this information is important it should be mentioned directly with the results, especially as different ages are used in Fig.3. (And exposing fish for 5 days to LD does not necessarily imply that the test age starts with 5dpf.).*

This information has been added to each of the figure legends.

*8.> Before the mansucripts is published it MUST add information on light intensity and spectrum in its supplementary material. This information is absolutely critical when presenting results on photoreceptors.*

This information is embedded in the material and methods. We have added precisions to our initial version as we agree that such information are crucial both to the understanding of the system and the reproducibility of the data **(**l 828-831**).**

*9> Based on the phase entrainment experiments with the lak mutants, the authors conclude that “Retinal Ganglion Cells are differentially required in a phase delay and a phase-advance paradigm of photoentrainment”. This interpretation needs some revisions. The authors cannot really interpret their results as a test for the impact of RGCs. lak mutants are very differently pigmented compared to their wildtype counterparts, which implies that less light will be able to reach all the tissues of the mutant fish compared to wildtype siblings.
Also, where is ath5, the gene mutated in lak mutant fish, expressed besides the eyes? This should be mentioned and considered for the interpretation of the results.
A possible way to discriminate between RGC vs. pigment vs. ath5 impacts on phase advance entrainment would be to repeat these experiments with wildtype vs. lak-/- eye-ablated larvae. However, the reviewer is aware that this will likely require a new animal experimental permit request. No matter the country- these legal permits usually take months to get. Thus, a vigorous discussion of the different interpretational alternatives would be an appropriate approach to address the raised concern, if such a permit does not already exist.*

We are grateful to the reviewer for this very interesting point. Indeed, *ath5* mutants are more pigmented. Along this line, surgical removal of the eye impairs the aggregation of pigment granules within pigment cells (Shiraki et al., 2010) which shows a role for the eye in body color changes. The reviewer suggest that this change could affect the way larval cells perceive light and suggest to perform surgical eye ablations. However, apart from ethical considerations (permit and laboratory practices), we would not trust experiments where larvae have undergone surgery as we expect that manipulation of the animals and their recovery at variable rates is likely to affect both their locomotor activity and the light-training. We therefore changed our conclusion to say that “absence of a functional eye differentially affects the induction of a phase delay and a phase-advance following pulses of light during the subjective night” and discuss the possibility that the role of the eye in the phase advance paradigm is indirect and linked to pigmentation.

Finally, we have added precisions regarding the expression of *ath5* which is specific to the retina and is expressed nowhere else (Masai et al., 2000), on p3: “ Phylogenetic tree analysis suggests that the zebrafish gene is an ortholog of Xath5, and so we designated this gene *ath5*. However, unlike the Xenopus gene, in situ hybridization analysis revealed that *ath5* is expressed exclusively in the developing retina”).

*10.> Another concern on the PA results is the aspect that is really not well visible in the averaged data from the three experiments. The authors address the issue by showing a single experiment in the supplemental, which is really helpful. It would be even more convincing if they include all three experiments individually in the supplemental materials.*

We have added a fourth experiment to our statistics, which further confirms the obtained result. We found out that the presence of larvae for which the Biodare FFT_NLLS method does not manage to extract a period between 18 and 30 hours and an associated phase is what obscures the data on the graph. This is in agreement with our preliminary observations showing that larvae are particularly sensitive to a pulse of light in the late subjective night which strongly disorganize their activity (see commentary). As supplemental data, we now show only the animals in which the curve fitting method was able to extract two phases one before the pulse and one after.

We hope this facilitates the reader work as the phenotype is much clearer on this supplemental graph.

*11. > The diel expression changes of opn4xa in interneurons of the eye other than RGC is interesting. A suggestion for improvements: This needs some rewording as RGCs are also interneurons. The anatomy and cell types of the vertebrate eye are very well described. The authors should be able to assign a more specific label to the cells- are these likely amacrine or horizontal or bipolar cells? This should be possible to tentatively assign based on position and cellular morphology. This provides valuable information for future studies.*

In our view, RGCs are projection neurons as they produce a long axon which project far away from the soma of these cells. As such, this is the classification we found in the literature. For instance, in (Sanes and Masland, 2015), on p.3: “In this nomenclature, there are five classes of neurons in the vertebrate retina: photoreceptors, which detect light; horizontal, bipolar, and amacrine cells, which are interneurons that process the output of photoreceptors; and RGCs, whose axons convey visual information to the rest of the brain through the optic nerve. The neurons are arranged in three cellular (nuclear) layers, separated by two synaptic (plexiform) layers”. In Figure 1A from (Kölsch et al., 2021), the scheme clearly differentiates the interneurons from the RGC making it clear that RGC do not belong to the interneuron category.

Based on position, we think the *opn4xa* + cells of the interneuron layer are most likely horizontal and amacrine cells. We have added this information in the legend of FigureS3.

12.> *The difference in the fos staining is very interesting, as it indeed suggests that much of the pineal light perception is via opn4xa during these larval stages. This is remarkable given many opsins are present in the fish pineal. This could be given some emphasis in the text.*

As answered to comment #6, *opn4xa* is only responsible for the LIGHT ON response of the pineal, we do not expect the LIGHT OFF response to be affected in the pineal as there are numerous other opsins expressed in the pineal (Cau et al., 2019).

*13.„Similarly to the analysis we performed in lak larvae, for each of these condition“
> Should be „similar“.*

l. 421. This has been corrected.

*Discussion:

14."Melanopsin expressing cells of the eye are the sole mediators of light input to the circadian rhythm in
nocturnal mammals."> ipRGCs are also considered the sole mediators of entrainment by light in humans- i.e. diurnal mammal. Please rephrase the sentence.*

The sentence has been rephrased : “Experiments performed in mouse have established a role for melanopsin expressing cells of the eye in mediating light input to the circadian rhythm in mammals”..on l 636

15.>*It would be important really interesting to see how the authors embed their work into the current general knowledge on Opsin functions in teleost fish. There are many more and many even described to be expressed in the pineal. In this context the fos expression comparison between mutant and wildtype is particularly remarkable. Do the authors expect that this sole role of opn4xa changes with later stages of development? What’s known about possible alterations of opsin functions in teleosts? Or do they think that this is due to the specific spectrum/intensity of light they use?*

There are indeed 42 opsins in zebrafish (Davies et al., 2015), among which *exorhodopsin, red cone opsin*, *parietopsin, parapinopsin1* and *parapinopsin2* are expressed in the developing pineal gland (Cau et al., 2019; Koyanagi et al., 2015; Wada et al., 2018). To the best of our knowledge, no mutant has been reported for these opsins. Morpholino experiments suggest that in absence of *exorhodopsin* function, transcription from the melatonin synthesis gene serotonin N-acetyl transferase2 (*aanat2*) is decreased but it is not clear how this impacts locomotor activity (Pierce et al., 2008). Finally, the only opsins mutant that have been generated in zebrafish are *opn4.1* and *opn4xb* and the double mutant does not show a circadian phenotype as explained in the discussion (l 766-767). Altogether we have already summarized in our paper the most salient facts concerning opsin functions in zebrafish as unfortunately not much is currently known.

*16. >The argument with the “destabilized” clock is cryptic. First, what exactly LL is doing depends very much on its intensity. This information is entirely lacking (see above). Second, moderate LL will cause differences in period length (see Aschoff, J. Cold Spring Harb Symp Quant Biol. 1960; 25:11-28, but besides the mentioned examples that’s also established for humans, the insect Drosophila, the annelid Platynereis). The mentioned “destabilization” likely refers to an effect seen under strong LL when it is assumed that this causes a constant re-setting of the circadian phase and hence desynchronization of the cellular networks and ultimately behavior.
One possible alternative explanation for the observed effects on the LL period of opn4xa mutants is that they sense less light (i.e. less light reaches the fish’s circadian clockwork) and hence– consistent with the above mentioned observations in many organisms– mutants show a slight period length difference.*

The information concerning light intensity is embedded in the material and methods, the light we are using for the LL experiment (20 lux) is not strong, however, we have observed that under such conditions the rhythms of locomotor activity dampens much faster than in DD (Reviewer figure 1); this is what we were mentioning under the term destabilization of the clock. It is indeed not entirely clear if it involves similar mechanisms to what is observed in Drosophila or the other species the reviewer mentions. We have modified the discussion accordingly and also introduced the other explanation mentioned by the reviewer.

Reviewer Figure 1: Average distance moved from n=58 ctl larvae. The mean is represented in 10 min bins after one round of smoothing each using ten successive time points was applied

**Reviewer #2:**

Please bear in mind that the line numbers correspond to the manuscript with highlighted changes and not the ‘text only’ version.

*1. Even though the circadian functions of intrinsically photosensitive retinal ganglion cells (ipRGCs) have been most revealed in nocturnal mice. Studies have shown that diurnal rodents, even humans, likely possess functional equivalent ipRGCs as nocturnal mice (doi:10.1371/journal.pone.0073343, doi.org/10.3389/fneur.2021.636330). Thus, in the title, as well as numerous descriptions and discussions in the manuscript, the authors should consider the photoentrainment differences between diurnal zebrafish and nocturnal mice carefully, as these differences are not just between diurnal zebrafish and nocturnal mice.*

The papers quoted by the reviewer describes the existence of melanopsin expressing RGCs in the retina of human, macaques as well as in the diurnal rodent *Arvicanthis ansorgei* . However, while these cells clearly share morphological, electrophysiological and transcriptomics features with the murine ones, evidence that they have a function in circadian rhythms is, to the best of our knowledge, still lacking. We have rewritten the introduction according to the reviewers comments:

L70-71 : “Similarly, in human, fifty percent of blind people exhibit circadian misalignement with the LD cycles (Hartley et al., 2018; Lockley et al., 2007; Sack et al., 1992)”.

L 107-110 : “ipRGCs are well established to mediate circadian and direct effects of light on behaviour in nocturnal mammals. Based on the conservation of the nervous system, observations made in human blind people and the description of ipRGC populations in the diurnal rodent Arvicanthis ansorgei as well as in human, ipRGCs are considered to play a similar role in diurnal mammals (Karnas et al., 2013; Mure, 2021)”.

*2. The authors should examine possible mechanisms that underpin what they observed in locomotor activities. For instance, the expression of two light-inducible circadian clock genes, per2 and cry1a(a), in lak and opn4xa mutants should be done.*

Concerning the *lakritz* mutant, we observed a locomotor phenotype upon a pulse of one hour of light at CT21. According to this locomotor phenotype, we expected less induction of light-induced genes in *lak* larvae compared to siblings at CT22 after a pulse of one hour of light. We have cloned a *per2* probe and analyzed the expression of this gene at CT22 either in animals maintained in the dark or submitted to a one hour pulse of light (reviewer figure 2). This analysis did not reveal any induction of *per 2* in response to light at this stage. In contrast, we have observed induction of *per2* in the brain of siblings and *lak* larvae after a pulse of light performed at CT16. However, showing this data will require precise mapping of the brain areas where this induction occurs and we think this is beyond the scope of this study given that neither *opn4xa* -/- nor *lak* -/- larvae show a phenotype in this paradigm.

The induction of *per2* by light at CT16 but not at CT21 is reminiscent of what happens in mice where *per2* and controls phase delays but not phase advances (Albrecht et al., 2001) and *per2* is not induced in the rat SCN upon a light pulse administered during the late subjective night (CT 20; (Miyake et al., 2000)) . Finally at CT 22, the *per2* expression pattern was very similar between *lak* and sibs both in the dark and after a pulse of light. Therefore, there is currently no candidate molecular mechanism for the effect of the *lakritz* mutation regarding the induction of a phase advance. We think solving this question will require further exploration and is beyond the scope of this study.

Reviewer Figure2: *per2* is expressed in the telencephalon of *lakritz* larvae and siblings independently of the administration of a light pulse at CT21. A *per2* probe encompassing the entirety of the second exon was cloned by PCR using the following oligos: Forward primer: 5’-AAATCCGAGTGTCCGTCTGC-3’, Reverse primer with T7: 5’-TAATACGACTCACTATAGGGTCTTGTTGCTTCCCGATGAC-3’; before fixation for *in situ* at CT 22 the larvae were treated exactly like in the experiment described in Figure 2D, meaning that they were not depigmented before the light pulse which was administered from CT21 and CT22 (CT 22 pulse). In parallel, larvae from the same litter were maintained in the dark and fixed at CT22 (CT 22 dark). The white ellipse identifies the pineal gland where no expression is observed. Scale bar: 50 μm.

When maintained in LL, *opn4xa* -/- larvae show both an hyperactivity phenotype during the first night as well as a decrease in period. We performed a thorough analysis of clock genes expression using RTqPCR which is now included in the paper (Figure 5). It revealed subtle alterations whose relationship with the molecular phenotypes is now discussed.

3*. The authors should use rhythmicity programs, such as Hughes et al. 2010. JTK_CYCLE: an efficient nonparametric algorithm for detecting rhythmic components in genome-scale data sets. J. Biological Rhythms) and/or MetaCycle (Wu et al. 2016. MetaCycle: an integrated R package to evaluate periodicity in large scale data. Bioinformatics) to analyze their behavioral data, which produces, rhythmicity probability, period, amplitude, and phase of each data set. For instance, in Figures 1C and E, it may have differences in amplitudes and phases between lak and sib*

We have performed such an analysis using the biodare platform (Zielinski et al., 2014) which analyzes all the parameters requested by the reviewer. We observed no difference in the amplitudes as shown in reviewer Figure3.

Reviewer Figure3: Z-score of the amplitude of circadian rhythm in control and *lak* (A,B) or *opn4xa-/-* (C,D) larvae in DD (A,C) or LL (B,D). Estimation of the amplitude was generated using the FFT-NLLS method. Each grey point represents a single larva. We did not observe any statistical difference between mutant and control in any of the conditions using t-tests.

To obtain rhythmicity probability, we calculated the percentage of larvae in which an appropriate period (comprised between 18 and 30 hours) was successfully extracted. The results are summarized in reviewer table 1 and table2.Overall, the *opn4xa* and *lak* mutation do not impair the rhythmicity of the larvae in DD and LL.

| condition | % of rhythmic animals |
| --- | --- |
| Ctl DD | 96,4% |
| *lak* DD | 97,5% |
| Ctl LL | 96,3% |
| *lak* LL | 98,8% |

Table 1: Percent of rhythmic *lak* and sib larvae in DD and LL; The difference between the ctl DD and the *lak* DD populations is not significant using a khi-two test p= 0.957, similar results are obtained between the ctl LL and the *lak* LL, p=0,91.

| condition | % of rhythmic animals |
| --- | --- |
| WT DD | 98,5% |
| *opn4xa-/-* DD | 92,8% |
| WT LL | 98,5% |
| *opn4xa -/-*LL | 98,5% |

Table 2: Percent of rhythmic *opn4xa -/-* and ctl larvae in DD and LL. The difference between the WT DD and the *opn4xa*-/- DD populations is not significant using a khi-two test, p= 0.06

*4. In Figure 1, why D used “four complete cycles in DD” while D used “three complete cycles in DD”?*

We filmed four cycles for the DD experiments versus three for the LL experiments as we observed that in LL the fourth cycle was extremely dampened and did not help in the period calculations.

*5. In the two-pulse skeleton photoperiod experiments, why a two-hour pulse of light at CT 16 while a one-hour pulse of light at CT21? What is the rationale?*

We have observed that a pulse of two hours of white light at CT 21 strongly disrupts the circadian rhythm which is why we use only one hour at this stage. On the other hand at CT 16 a pulse of one hour of light is not sufficient to induce a phase shift. These differences in the sensitivity of the circadian system depending on circadian time are very interesting but we think it is beyond the purpose of the current paper to study this.

6. *What machine was used to conduct behavioral assays? Was it homemade?*

Yes this is a homemade machine that is identical to the one described in (Vignet et al., 2013) .We now describe it a bit more in the material and methods section (l 830-831, see below in comment 8).

7. *As most of the behavioral assays were done after 6 day post fertilization, when larvae start foraging, were these larvae fed?*

The larvae are not fed. A device to feed each larvae in each of the 96 wells without modifying the illumination does not exist to the best of our knowledge.

8. *What is the lux (intensity) of light used?*

This information is embedded in the material and methods L 828-831 : “Light intensity during the entrainment was 110 lux at 6500K. On the evening (16:00-20:00), the plate was put in an hermetic box in a dark room maintained at 27°C with a heater. This home-made box (Vignet et al., 2013) was continuously illuminated from below with two panels of infra-red lights as well as neutral white light (4000K, 20 lux at water surface))”

9. *Δphase calculated via the difference of phase between the two last days (“after the light pulse”) and the two first days (“before the light pulse”) may not be accurate because of the developmental stage-specific effects. Δphase should be the phase difference between larvae with the light impulse and larvae without the light impulse of the same period.*

We are not aware of any stage-specific effect on the phase. On the contrary, we noticed that in constant darkness, the phase is delayed of one hour at each cycle which perfectly matches the expectation for a cycle bearing a 25 hours period (table 1-3). The calculation proposed by the reviewer is incompatible with a statistical analysis as there is no way we can have the same animals at the same age maintained in the dark and light pulsed at the same time. This means that only a population level difference can be calculated and no statistics can be performed on such a difference in the average phases. On the contrary our system permits to have the difference before and after the pulse for each animal and therefore enables statistical analysis. Finally, we compare the phase difference induced between the pulsed animals and the animals kept in the dark at the population level.

*10. It would be better to show the data in Tables 1-3 as Figures.*

We would completely agree with this comment if we were measuring phases. However our focuss is phase differences for which there is no specific graphical representation available. A solution would be to use box plots but we do not see that it is preferable to the numbers in a table.

*11. In Figure 3, B, C, and D should have scale bars. Quite contrary to what is claimed in the manuscript, I see differences in the numbers of positive (opn4xa and c-fos) cells, as well as their spatial structures between opn4xa mutant and wild-type control larvae.*

Scale bars have been added as requested.

We do not really understand the reviewer’s comment concerning the number of *opn4xa*+ and *fos*+ cells. In the eye, the *opn4xa*+ cells have been counted and no difference is observed between the genotypes: RGC layer : *opn4xa* +/+: 26.17 ± 7.33, n= 6; *opn4xa*-/-: 23.8±6.92, n=10; p=0.42 using a Mann-Whitney two-tailed test; interneurons layer : *opn4xa* +/+: 26.33± 11.5 ; *opn4xa*-/-: 22.9±7.19; p=0.63 using a Mann-Whitney two-tailed test. Concerning the pineal, similar counts were made which show no difference for the number of *opn4xa*+ cells : *opn4xa* +/+: 4.33±1.51 n=6, *opn4xa*-/- : 4.75± 1.98 n=9, p=0.85 using a Mann-Whitney two-tailed test; while *fos*+ cells are absent/reduced in the *opn4xa* -/- background : at 3 days *opn4xa* +/+: 4.8 ± 0.8 (n=9), *opn4xa-/-*: 0.25 ± 0,7 cells (n=8) per pineal; at 7 days *opn4xa* +/+: 5.9 ± 3,5 (n=9) *opn4xa-/-*: 0.4 ± 0,7 cells (n=13); mean ± S.D All these numbers are indicated in the paper. If the comment relates to the aspect of the pineal gland in the pictures, in the precise pictures that are shown, we counted 7 *opn4xa*+ cells for the WT and 8 for the *opn4xa* -/- mutant (Figure 3C). The cells are not exactly arranged the same in the WT and *opn4xa* -/- sample but this variation does not exceed the one observed between two samples of the same genotype and it is impossible to obtain two samples with exactly the same distribution. Concerning *fos* we counted 3 cells in the control and none in the mutant which reflects well what is observed in the general population.

*12. Where is the tcf7 expression data?*

The pictures illustrating the numerical data introduced in the text are now shown as Figure S4.

*13. No figure legend for supplementary figure 3. Scale bars should be shown in A-D.*

Both are now added.

*14. Put “To genotype opn4xa individuals, we used a classical PCR protocol with the following oligos: 5'-
GGACGCCTCCAAACTTC-3' (Forward) and 5'-CGAACACCCACTCCTTGTAC-3' Reverse). PCR products of different sizes were obtained (110bp for the wt allele and 127bp for the mutant allele) and resolved on a 4% agarose gel” into “Generation of an opn4xa mutant allele”.*

This modification was done.
 *15. “Enucleated mice” should be “eye enucleated mice”?*

This modification has been done (l. 68).

16. “*Mice mutant for Opn4 show” should be “Mice mutant for Opn4 shows”*

This modification has been done (l.75)

17. “*In contrast, mice with no ipRGCs or with impaired neurotransmission from ipRGCs show no entrainment to LD as well as no phase delay following a light pulse at CT16 (Cahill, 1996; Güler et al., 2008; Kofuji et al., 2016).” Is Cahill, 1996 relevant here?*

Indeed, this is a mistake that we corrected.

**Reviewer #3:**

Please bear in mind that the line numbers correspond to the manuscript with highlighted changes and not the ‘text only’ version.

1) *For a number of reasons, I find the title somewhat misleading: The word „profound“ is an extremely strong word and I do not agree it is appropriate for the findings that have been reported here. Also, the reference to diurnal and nocturnal implies that somehow the results have ascribed special significance to the temporal niche occupied by these two species. I don’t see this.*
The title has been changed for something more neutral: “Contribution of the eye and of *opn4xa* function to circadian photoentrainment in the diurnal zebrafish”

*2) First sentence of abstract (as well as first sentence of Results): the importance of the eye for controlling circadian rhythms is not a special feature limited to „nocturnal rodents“ (- the inference here). It is also very clearly the case for other diurnal vertebrates such as humans. This recurrent reference to diurnality and nocturnality throughout the text needs to be toned down.*

The reviewer is right that there are reports in human showing that blind people have circadian deficits. These are now listed in the introduction : ‘Similarly, in human, fifty percent of blind people exhibit circadian misalignement with the LD cycles (Hartley et al., 2018; Lockley et al., 2007; Sack et al., 1992)’. We have changed the first sentence of the abstract: “The eye is instrumental for controlling circadian rhythms in mice and human”. Similarly, the first sentence of the results section is now: ‘ Photoentrainment in mouse and human requires a functional eye’

*3) There is already much evidence for major differences between the organization of the zebrafish and mouse circadian timing systems, so the last sentence of the abstract (as well as the title) which implies that this is somehow the original conclusion of this work is also misleading.*

The title has been completely changed and the last sentence of the abstract tuned down: “Our results support the idea that the eye is not the sole mediator of light influences on circadian rhythms of locomotor activity and highlight differences in the circadian system and photoentrainment of behaviour between different animal models”. We think that the word support does not imply that this is something original but rather that it reinforces previous observations which correspond to the situation. On the other hand, as previous papers describing this difference in organization of circadian systems (especially from the Foulkes and Whitmore lab) have not addressed the role of the eye regarding locomotor activity rhythms, we insist on this aspect in the last sentence (see answers to reviewer 1 comments 3 and 5, below).

Reviewer 1 comment 3:

3. „*raising the intriguing possibility that vertebrates might employ different molecular/cellular circuits for photoentrainment depending on their phylogeny and/or temporal niche.“
> It is already well established that in zebrafish different peripheral cell types are directly photosensory. See work by the labs of David Whitmore, Nick Foulkes etc.. The authors even refer to the relevant literature in their introduction.
So, this is not raising a possibility, but it is a long-standing fact. The question is more to the mechanistic details, how different photoreceptors contribute to it, and why this ability apparently got lost in mammals. This is where the authors actually make their contributions. They might be not so obvious, but they are nevertheless important for the large picture of non-visual photoreceptor functions. So, it would be appropriate to re-word this summary accordingly.*
As mentioned by the reviewer and as detailed in the introduction, zebrafish peripheral cells are photosensitive and this sensitivity contributes to the entrainment of the molecular clock as shown by the Whitmore and Foulkes lab. The biological role of this peripheral photosensitivity is however unclear. For instance, it is possible that this peripheral photosensitivity, does not impact the photoentrainment of behavior but serves local functions. On the other hand, one could imagine that a behavior would require some level of central control and would be difficult to pilot from the periphery.

The sentence that the reviewer refers to specifically deals with the behavioral part of the circadian rhythms and not the molecular clock, which might not have been clear enough in our manuscript, as our point is to address the role of the eye and of the *opn4xa* photosensitivity of the pineal regarding photoentrainment of locomotor activity**.** We however agree that the sentence we used is too strong and tunned it down (l.54-56) to say that it **supports** rather than **raise** that “vertebrates might employ different molecular/circuits for photoentrainment of behavior depending on their phylogeny and/or temporal niche”.

Reviewer 1 comment 5:

*5."Although ipRGCs mediate circadian and direct effects of light on behaviour in nocturnal mammals, it is unclear if these roles are conserved in diurnal and non-mammalian species"*
*>The statements after these sentences need serious rewording. It is long established especially by the work of David Whitmore, Nicholas Foulkes and their labs that individual cells in fish are themselves light sensitive, independent of the eyes. The authors refer to Cavallari et al.. 2011, but do not embed the findings and conclusions of this paper well into their introduction. Directly after the citation they write: “While the function of the eye regarding circadian rhythms has not been addressed in fishes…” and “The role of this local photodetection regarding circadian behaviours has not been explored… “.
However, Cavallari et al.. 2011 explicitly states (in their summary!): “We reveal that eye loss does not account for this “blind” clock. Specifically, mutations of two widely expressed non-visual opsin photoreceptors (Melanopsin and TMT opsin) are responsible for the blind clock phenotype in the cavefish.”*

*Cavallari et al.. 2011 provides evidence for this statement by tissue culture experiments. Interestingly, Cavallari et al.. 2011 have not performed the lak- mutant experiments, done in this manuscript. So, the current study certainly adds information, but the precise value of these new experiments needs to be better worked out. Thus, the current introduction needs serious rewording.*

Indeed, Cavallari et al., 2011 have shown that the blind cavefish Phreatichthys andruzzii bears mutations in *opn4m2* and *tmt* opsin,which are predicted to produce non- functional proteins in this species and that reexpression of the non-mutated (zebrafish) forms in a cell line they derived from cavefish fin clips rescues the rhythmic light-induced expression of a *zfper2: luciferase* reporter (Fig.7 and 8). What **they did not show** is that re-expression of the non-mutated forms of these opsins in the animal rescues photoentrainment of their locomotor activity. Thus, when in their summary they refer to the “blind clock”, in our opinion, they are talking about the molecular clock of fin cells. While these findings are extremely important, it is important to note that, in contrast to the Cavallari analysis, we focuss on the control of behavior and locomotor activity, we have tried to make this issue clearer for the reader and modified the text accordingly: l.111 to 133:

“While the zebrafish has emerged as a powerful non-mammalian diurnal vertebrate model for chronobiology, the neural circuit controlling photoentrainment of behaviours has not been identified in this species. The observation that two species of cavefishes bearing eye degeneration *: Astyanax mexicanus* and Phreatichthys andruzzii do not show robust light-entrainable circadian locomotor activity rhythms could suggest that eye function is crucial for photoentrainment of locomotor rhythms in fishes (Cavallari et al., 2011). However, the observed circadian locomotor phenotypes could result from additional photoreceptive deficits. Indeed, Phreatichthys andruzzii present mutations in several opsin genes that are expressed in extra-ocular locations (Cavallari et al.. 2011). While the function of the eye regarding circadian rhythms of behaviours has not been addressed in fishes, in the zebrafish model, an additional level of complexity arises from the observation that all cell types are photosensitive (Carr and Whitmore, 2005; Vallone et al., 2004; Whitmore et al., 2000) . This local photodetection could serve local functions (metabolism, transcription, cell cycle) or participate to the control of behaviors, although some level of central control is expected for orchestrating a complex process such as behavior. As such, the relative importance of central versus peripheral control for the photoentrainment of locomotor activity is still unclear”.

*4) First sentence of the Introduction: „regulate a phenomenal variety of biological functions“ is once again a wild over statement!*

The word phenomenal was removed (l.61).

*5) End of second paragraph of the Introduction section. „…which is though to behave as a ‚master clock‘….“ is a little understating the issue. There is so much data to support this notion that I think it would be more accurate to state „…..is well established to serve as a master clock”.*

This was changed on l. 98: “This hypothalamic nucleus functions as a ‘master clock’ that synchronizes peripheral clocks present everywhere in the body”.

*6) Introduction, paragraph 4: While the cavefish P. andruzzii does not show significant locomotor activity rhythmicity under LD cycle conditions (Cavallari et al., 2011), Astyanax mexicanus Pachon (blind form) does show rhythmicity under artificial LD cycles (Beale et al., 2013). This statement should be corrected. Last sentence, „peripheric“ should be corrected to „peripheral“, and „………still begs for an answer“ should be corrected to „…. is still unclear“*
l. 132/133 The sentence has been modified as required.

*Results and Conclusions*

*1) The authors should provide a more detailed description of the Lakritz mutant retinal phenotype as well as details of the underlying mutation in Ath5 etc.*

We have completed our description of the *lakritz* mutation as requested l 161-164 : “The gene mutated in *lak*, encoding for the bHLH transcription factor ATOH7 (ATH5) is expressed in the developing retina and nowhere else (Masai et al., 2000). The lak^th241^allele we used bears a point mutation functions as a null allele which result in a total absence of both RGCs and a neural connection between the eye and the brain (Kay et al., 2001)".

2) *2nd paragraph: „The reduction in activity observed during the day in LD conditions thus does not reflect a defect in circadian control“ is not an accurate statement based on the data that this statement is based on. Better would be „The reduction in activity observed during the day in LD conditions thus does not affect the persistence of rhythmicity under free running conditions in DD“.*

The sentence has been modified exactly as requested.

*3) The opn4xa knock out experiments completely lack formal proof that the mutation indeed does lead to production of a significantly truncated and non-functional protein!!!!*

In absence of a specific antibody for OPN4XA this question is difficult to address. This is the case for most zebrafish papers involving mutant alleles (see for instance (Chen et al., 2017; Ruggiero et al., 2021)).Indeed, most zebrafish papers in which a mutant line is generated rely on protein prediction. We see two things that could happen that would make the prediction false. A) The first is the use of **an alternative ORF:**

1. We have verified that all three ORFs generate a truncation from the mutated allele (stop codons are indicated with a star *)

First ORF MEESKDMDRGFFRKVDVPDHAHYIVAFFVAVIGALGVIGNLLVMYAFFSNKKLRTPPNYFIMNLAVSDFIRQ*

Second ORF

WRSRRIWIEAFSGKWTFLIMHTT*

Third ORF

GGVEGYGSRLFQESGRS*

2. Since we noticed that there is a second ATG, present in both the wt and the mutant cDNA highlighted here:

atggaggagtcgaaggatatggatcgaggctttttcaggaaagtggacgttcctgatc**atg**cacactacatagtggcgttttttgtggccgtcatcggggctctaggagtgatcggaaacctgctggtcatgtacgccttctttagtaacaagaagctgc…

we verified that using this one as well leads to a truncated protein from the mutant allele

MDRGFFRKVDVPDHAHYIVAFFVAVIGALGVIGNLLVMYAFFSNKKLRTPPNYFIMNLAVSDFIRQ*

B) The mutation generates an **alternative splicing**: To verify that this is not happening, we have used a deep-learning-based website that predict splice acceptors and donors ([Spliceator (lbgi.fr)](http://www.lbgi.fr/spliceator/)). For both the wt and the mutant genomic sequence, we submitted the three first exons on the website, which yielded the following predictions: for the wt allele:

For the mutant allele :

The mutation is located at position 10971 on our genomic DNA fragment which results in the last acceptor site to be at a different position in the wt and the mutant. Apart from this the algorithm does not detect the appearance or diseappearance of donor/acceptor sites in the mutant allele.

Altogether, we do not see a reason why the prediction from the mutant allele could fail. If the reviewer as an alternative that we did not think of, we will be happy to analyze it. In the end, we make it clear in the result section that the mutation is predicted to be non-functional and that in absence of a good antibody the formal proof of this is lacking*.*

4) *Light-induced fos expression in the pineal gland is used as a proxy for „intrinsic photosensitivity in pineal cells“. However, this assumes that there is just a single pathway linking Opn4xa with gene expression. I am not convinced that there is sufficient data to support such a hypothesis. Therefore, it would be important to test expression of other light-regulated genes such as 6-4 photolyase, Cry1a or Per2 in these pineal cells.*

We have looked at the induction of *per 2* after light pulses at CT 16 and CT21 (the results are described in the reviewer2 comment 2 section, see below) but we did not observe any induction of *per2* in the pineal area. More generally, it is not clear to us why the reviewer thinks that induction of *per 2*, *cry1a* or *6-4 photolyase* would necessary reflect a direct activation by light as for instance *per* genes are also induced in the murine SCN and this effect is thought to be mediated by ipRGC projecting onto this structure. In addition, our goal when using *fos* expression was to use it as a marker for neuronal activity rather than looking at pathways linking OPN4XA to gene expression. On the other hand, we agree that our conclusion that *opn4xa* photosensitivity is lost in the mutant might be too strong and tunned it down. Our conclusion is now as follows: “We therefore used the *opn4xa* mutant as a model in which the pineal ON-response is impaired”.

reviewer2 comment 2:

*2. The authors should examine possible mechanisms that underpin what they observed in locomotor activities. For instance, the expression of two light-inducible circadian clock genes, per2 and cry1a(a), in lak and opn4xa mutants should be done.*

Concerning the *lakritz* mutant, we observed a locomotor phenotype upon a pulse of one hour of light at CT21. According to this locomotor phenotype, we expected less induction of light-induced genes in *lak* larvae compared to siblings at CT22 after a pulse of one hour of light. We have cloned a *per2* probe and analyzed the expression of this gene at CT22 either in animals maintained in the dark or submitted to a one hour pulse of light (reviewer figure2). This analysis did not reveal any induction of *per 2* in response to light at this stage. In contrast, we have observed induction of *per2* in the brain of siblings and *lak* larvae after a pulse of light performed at CT16. However, showing this data will require precise mapping of the brain areas where this induction occurs and we think this is beyond the scope of this study given that neither *opn4xa* -/- nor *lak* -/- larvae show a phenotype in this paradigm.

The induction of *per2* by light at CT16 but not at CT21 is reminiscent of what happens in mice where *per2* and controls phase delays but not phase advances (Albrecht et al., 2001) and *per2* is not induced in the rat SCN upon a light pulse administered during the late subjective night (CT 20; (Miyake et al., 2000)) . Finally at CT 22, the *per2* expression pattern was very similar between *lak* and sibs both in the dark and after a pulse of light. Therefore, there is currently no candidate molecular mechanism for the effect of the *lakritz* mutation regarding the induction of a phase advance. We think solving this question will require further exploration and is beyond the scope of this study.

Reviewer Figure2: *per2* is expressed in the telencephalon of *lakritz* larvae and siblings independently of the administration of a light pulse at CT21. A *per2* probe encompassing the entirety of the second exon was cloned by PCR using the following oligos: Forward primer: 5’-AAATCCGAGTGTCCGTCTGC-3’, Reverse primer with T7: 5’-TAATACGACTCACTATAGGGTCTTGTTGCTTCCCGATGAC-3’; before fixation for *in situ* at CT 22 the larvae were treated exactly like in the experiment described in Figure 2D, meaning that they were not depigmented before the light pulse which was administered from CT21 and CT22 (CT 22 pulse). In parallel, larvae from the same litter were maintained in the dark and fixed at CT22 (CT 22 dark). The white ellipse identifies the pineal gland where no expression is observed. Scale bar: 50 μm.

5) *In the discussion, the authors refer correctly to the large opsin gene repertoire of zebrafish and how this might complicate the interpretation of their results. Given this central issue, it is important that the authors defend in more detail precisely why this particular opsin was chosen for the loss of function experiments. Furthermore, it might be valuable for the reader to already mention this issue in the Introduction..*

*Opn4xa* was chosen based on the fact that it is the only opsin we know that is expressed in the pineal LIGHT-ON cells. As in mice, the extrinsic photosensitivity of melanopsin drives and ON-response in ipRGC, and ipRGCs functioning in photoentrainment are expected to do so in an ON-fashion (McNeill et al., 2008), we used our *opn4xa* mutant to address the role of the pineal ON response. This is now detailed in the introduction:

L 144-146 : “Interestingly these *opn4xa*+ projection neurons function in a LIGHT ON fashion while *opn4xa*- projection neurons function in a LIGHT OFF manner (Sapède et al., 2020) . We engineered an *opn4xa* mutant in which the LIGHT ON response of the pineal is impaired”

We hope that both reviewers and editors will now find the manuscript suitable for publication.

Sincerely,

On behalf of the coauthors,

Dr Elise CAU

**REFERENCES**

**Albrecht, U., Zheng, B., Larkin, D., Sun, Z. S. and Lee, C. C.** (2001). MPer1 and mper2 are essential for normal resetting of the circadian clock. *J. Biol. Rhythms* **16**, 100–104.

**Carr, A.-J. F. and Whitmore, D.** (2005). Imaging of single light-responsive clock cells reveals fluctuating free-running periods. *Nat. Cell Biol.* **7**, 319–321.

**Cau, E., Ronsin, B., Bessière, L. and Blader, P.** (2019). A Notch-mediated, temporal asymmetry in BMP pathway activation promotes photoreceptor subtype diversification. *PLoS Biol.* **17**, e2006250.

**Cavallari, N., Frigato, E., Vallone, D., Fröhlich, N., Lopez-Olmeda, J. F., Foà, A., Berti, R., Sánchez-Vázquez, F. J., Bertolucci, C. and Foulkes, N. S.** (2011). A Blind Circadian Clock in Cavefish Reveals that Opsins Mediate Peripheral Clock Photoreception. *PLoS Biol.* **9**,.

**Chen, S., Reichert, S., Singh, C., Oikonomou, G., Rihel, J. and Prober, D. A.** (2017). Light-Dependent Regulation of Sleep and Wake States by Prokineticin 2 in Zebrafish. *Neuron* **95**, 153-168.e6.

**Davies, W. I. L., Tamai, T. K., Zheng, L., Fu, J. K., Rihel, J., Foster, R. G., Whitmore, D. and Hankins, M. W.** (2015). An extended family of novel vertebrate photopigments is widely expressed and displays a diversity of function. *Genome Res.* **25**, 1666–1679.

**Hartley, S., Dauvilliers, Y. and Quera-Salva, M.-A.** (2018). Circadian Rhythm Disturbances in the Blind. *Curr. Neurol. Neurosci. Rep.* **18**, 65.

**Kölsch, Y., Hahn, J., Sappington, A., Stemmer, M., Fernandes, A. M., Helmbrecht, T. O., Lele, S., Butrus, S., Laurell, E., Arnold-Ammer, I., et al.** (2021). Molecular classification of zebrafish retinal ganglion cells links genes to cell types to behavior. *Neuron* **109**, 645-662.e9.

**Koyanagi, M., Wada, S., Kawano-Yamashita, E., Hara, Y., Kuraku, S., Kosaka, S., Kawakami, K., Tamotsu, S., Tsukamoto, H., Shichida, Y., et al.** (2015). Diversification of non-visual photopigment parapinopsin in spectral sensitivity for diverse pineal functions. *BMC Biol.* **13**, 73.

**Lockley, S. W., Arendt, J. and Skene, D. J.** (2007). Visual impairment and circadian rhythm disorders. *Dialogues Clin. Neurosci.* **9**, 301–314.

**Masai, I., Stemple, D. L., Okamoto, H. and Wilson, S. W.** (2000). Midline Signals Regulate Retinal Neurogenesis in Zebrafish. *Neuron* **27**, 251–263.

**McNeill, D. S., Altimus, C. M. and Hattar, S.** (2008). Retina-clock relations dictate nocturnal to diurnal behaviors. *Proc. Natl. Acad. Sci. U. S. A.* **105**, 12645–12646.

**Miyake, S., Sumi, Y., Yan, L., Takekida, S., Fukuyama, T., Ishida, Y., Yamaguchi, S., Yagita, K. and Okamura, H.** (2000). Phase-dependent responses of Per1 and Per2 genes to a light-stimulus in the suprachiasmatic nucleus of the rat. *Neurosci. Lett.* **294**, 41–44.

**Pierce, L. X., Noche, R. R., Ponomareva, O., Chang, C. and Liang, J. O.** (2008). Novel functions for Period 3 and Exo-rhodopsin in rhythmic transcription and melatonin biosynthesis within the zebrafish pineal organ. *Brain Res.* **1223**, 11–24.

**Ruggiero, G., Ben-Moshe Livne, Z., Wexler, Y., Geyer, N., Vallone, D., Gothilf, Y. and Foulkes, N. S.** (2021). Period 2: A Regulator of Multiple Tissue-Specific Circadian Functions. *Front. Mol. Neurosci.* **14**, 718387.

**Sack, R. L., Lewy, A. J., Blood, M. L., Keith, L. D. and Nakagawa, H.** (1992). Circadian rhythm abnormalities in totally blind people: incidence and clinical significance. *J. Clin. Endocrinol. Metab.* **75**, 127–134.

**Sanes, J. R. and Masland, R. H.** (2015). The types of retinal ganglion cells: current status and implications for neuronal classification. *Annu. Rev. Neurosci.* **38**, 221–246.

**Sapède, D., Chaigne, C., Blader, P. and Cau, E.** (2020). Functional heterogeneity in the pineal projection neurons of zebrafish. *Mol. Cell. Neurosci.* **103**, 103468.

**Shiraki, T., Kojima, D. and Fukada, Y.** (2010). Light-induced body color change in developing zebrafish. *Photochem. Photobiol. Sci. Off. J. Eur. Photochem. Assoc. Eur. Soc. Photobiol.* **9**, 1498–1504.

**Vallone, D., Gondi, S. B., Whitmore, D. and Foulkes, N. S.** (2004). E-box function in a period gene repressed by light. *Proc. Natl. Acad. Sci. U. S. A.* **101**, 4106–4111.

**Vignet, C., Bégout, M.-L., Péan, S., Lyphout, L., Leguay, D. and Cousin, X.** (2013). Systematic screening of behavioral responses in two zebrafish strains. *Zebrafish* **10**, 365–375.

**Wada, S., Shen, B., Kawano-Yamashita, E., Nagata, T., Hibi, M., Tamotsu, S., Koyanagi, M. and Terakita, A.** (2018). Color opponency with a single kind of bistable opsin in the zebrafish pineal organ. *Proc. Natl. Acad. Sci. U. S. A.* **115**, 11310–11315.

**Whitmore, D., Foulkes, N. S. and Sassone-Corsi, P.** (2000). Light acts directly on organs and cells in culture to set the vertebrate circadian clock. *Nature* **404**, 87–91.

**Zielinski, T., Moore, A. M., Troup, E., Halliday, K. J. and Millar, A. J.** (2014). Strengths and Limitations of Period Estimation Methods for Circadian Data. *PLOS ONE* **9**, e96462.
