# Supplementary material for: Contribution of the eye and of opn4xa function to circadian photoentrainment in the diurnal zebrafish
Source: PLoS Genet. 2024 Feb 26;20(2):e1011172. doi: 10.1371/journal.pgen.1011172 (PMC10919856; doi:10.1371/journal.pgen.1011172)
Supplement: Attachment — Submitted filename: answertoreviewers.odt [file pgen.1011172.s027.odt]

Dear Dr Ewer and reviewers
We are submitting a revised version of our manuscript. We were happy that reviewer 1 and 3 were satisfied with our answers and addressed the minor revisions asked by reviewer3 and the comments of reviewer 2.
Please find enclosed a point-by point answer to these comments.

Reviewer #2:  
1. Regarding the Δphase, I suggested it should be the phase difference between larvae with the light impulse and larvae without the light impulse of the same period. Actually, it is the phase difference between those of the larvae under DD and PD/PA, which would be a better estimate.
The reviewer suggest that we calculate a Δphase between pulsed larvae and larvae maintained in the dark.  For achieving that, we need to obtain an average value of the ‘phase before’ (corresponding to the two first cycles of the experiment) for each condition. However, there are differences in the two first cycles between individual experiments of the same type in the controls. For instance, if we compare the PA experiments: 


Reviewer Figure1: A) Boxplot showing the phase of the first cycles (‘phase before’) between three PA experiments. Comparison between PA1 and PA2: p= 0.0006; PA2 and PA3 : p<0.0001, PA1 and PA3 :  p= 0,0957.  B) Boxplot showing the Δphase of three independent PA experiments. The Δphase calculated here is the phase after-phase before and corresponds to what we have represented in our manuscript.  Comparison between PA1 and PA2: p= 0,7361, PA2 and PA3 : p=0,4789, PA1 versus PA3, p= 0,2407.
	Given the interindividual and interexperimental variation, we chose to standardize the data using the difference between the final phase (‘phase after’) and the initial phase (‘phase before’), which is a common statistical procedure and leads to a parameter that is very stable between experiments (see Reviewer Figure 1B).
Finally, we want to highlight the fact that - in constant darkness, the phase is delayed of one hour at each cycle which perfectly matches the expectation for a cycle bearing a 25 hours period (table 1-3) and suggest that the developmental stage does not influence the phase
-we compare our Δphase between pulsed larvae and larvae maintained in the dark of the same age using Mann Whitney tests (table1-3) so the actual comparison that the reviewer asks for is actually performed but with a different method.
Finally, the raw data is available in the supplementary material section for anyone interested in using alternative statistical analysis methods.
2. Regarding the possible mechanisms underlying what you observed in locomotor activities, in particular, why “the eye/RGCs are dispensable for the induction of a phase delay following a pulse of white light at CT 16 but contribute to the induction of a phase advance upon a pulse of white light at CT21.” The parallel examination of the expression of two light-inducible circadian clock genes, per2 and cry1a(a), in lak mutant, should be done, rather than just examining the expression of circadian clock genes under LL conditions, as shown in Figure 5. The hypothesis could be that the expression of key circadian clock genes is altered in these light impulse schedules, which may underlie the behavioral phenotypes.
Since we observed a phenotype in the lak background with a statistically less important phase advance than in controls we addressed whether per2 and cry1a were induced upon administration of a one-hour light pulse at CT21 and how these inductions were affected by the absence of a functional lak gene. These results are described in figure 5.

3. Regarding what is shown in Figures 3B and 3C, the authors should select new representative panels that show similar numbers of opn4xa-positive cells in the mutant and wild-type controls.
We have selected other representative panels as requested (see Figures 4 and S4). Moreover, in addition to the numbers provided in the text, corresponding to cells counts in the different layers of the retina and the pineal of opn4xa+/+ and opn4xa-/- larvae, we have added corresponding histograms (Figure 4 B,C,E).

4. It is highly recommended that the authors should put some paramecia in each well, along with tested larvae, which should help obtain better behavioral data, avoiding having the data shown in Figure 1 B, where the third-day results show little difference between the mutant and wild types, likely due to the possibility that the larvae ran out of energy without feeding.
We thank the reviewer for this suggestion. Indeed, adding paramecia is a good idea to improve the locomotor performances of our fishes for long experiments. However, paramecia have a circadian rhythm of their own1,2 which we are afraid could interfere with our experiments as restrained feeding exert an entrainment effect3. Therefore ,while this could help for experiments aiming at looking at direct effects of light (ie masking), we fear it would interfere with studies on circadian rhythms and for this reason we do not use it here. We have added a comment in the legend of Figure1 to account for the possibility, suggested by the reviewer, that the absence of a lak phenotype during the third day in LD reflects the energy state of the larvae.

5. It is good that the authors performed the rhythmicity analysis of the relevant behavioral data with Biodare2. But the authors should cite the results, for example, locomotor activities of the lak mutant and wild type display rhythmicity with statistically significant P values. Put the P values on the respective panels.
We have incorporated the data on percent of rhythmic embryos (which was presented in our last letter of answer to reviewers) in the results section.

Minor issues:
In Figure 5. Should tefalpha be tefa? What is reverbB1? These genes should have IDs.
Tefalpha is indeed also called tefa and reverbB1 is nr1d2a, we have modified this in the Figures and have added the ZDB gene ID in the Supplemental table7 for the sake of clarity. 

Reviewer #3:  
1) Text modification 1.111-133
The circadian locomotor activity rhythm phenotype of Astyanax mexicanus is NOT documented in Cavallari et al, 2011. Instead this is refered to in the manuscript from the Whitmore group: Beale et al., 2013, Nature Communications. This citation should be added.
Reviewer3 is right, we have added Beale et al., 2013 for Astyanax work.

2) Text modification 1.111-133, Final sentence(s): “………..although some level of central control is expected for orchestrating a complex process such as behaviour. As such, the relative importance of central versus peripheral control for the photoentrainment of locomotor activity is still unclear.” Here, and in other parts of the manuscript, the authors tend to give the impression that if a central clock structure is involved in photoentrainment of complex behaviour, then it should operate like the mammalian SCN - so light input coming from the retina or other dedicated photoreceptors. However, as the authors state, the situation in fish is that most cell types, likely including neurons, are directly photosensitive. So, the division between central and peripheral clocks in fish is less well defined. Therefore, it is formally possible that a central CNS-based pacemaker could coordinate light entrainment of rhythmicity WITHOUT the need for indirect input from a functional photoreceptor - in some way acting more like a peripheral clock. This possibility should be raised to avoid any confusion.
The possibility that a central control of locomotor rhythms is caused by a directly photosensitive brain nucleus, addressed by reviewer 3, is indeed a good option we have added the following sentence “Finally given the widespread photosensitivity of the larval zebrafish, a central control could be exerted by a photosensitive brain nucleus in this species “ l.103-104 to include this relevant hypothesis.

3) Text modification
 1.161-164
The text should be corrected to “……. is expressed exclusively in the developing retina (Masai et al., 2000). The Lakth241 allele we used bears a point mutation and functions as a null allele which results in a total absence of both…….”

This was done (l134)

4) Concerning the lack of experimental evidence for the production of a truncated Opn4xa opsin protein.I apologise that I do not agree with the authors in their argumentation. Using the observation that most zebrafish papers in which a mutant line is generated do not show direct protein data is not a credible excuse. If a mouse-based project failed to provide such evidence then this would be considered as a serious negative point. I am sure that antibodies can be raised against proteins even from zebrafish - it just takes more time and effort!!! However, that said I do appreciate the authors arguments that protein prediction can be useful as indirect evidence that the mutation is likely to have been successful. As a compromise solution, I would advise the authors to state in the Results section text the lines of evidence that they have provided in their rebuttal letter, basically that they have eliminated the possibility of Alternative ORFs, Alternative ATGs and alternative splicing. At least this way, the reader can make up their own mind as to the confidence they can associate with the results obtained using this mutant.
We thank the reviewer for this suggestion. The requested explanations have been added to the text (l 302-305) and the sequences are now included in Fig S2 C-D.

5) The logic for requesting expression data for Cry1a, and 6-4 photolyase: These (as well as per2) have been extensively studied as light regulated genes, and the enhancers responsible for this photic regulation have been identified. There are also gene-specific differences in the mechanisms (TFs) whereby they are regulated by light. Furthermore, given the complexity of the opsin gene repertoire in the zebrafish, it seems likely that there is also complexity at the signalling and gene expression regulation levels of the light response. For this reason, analysing a small number of well characterized light - regulated genes would provide more compelling evidence for whether a particular cell type is truly photosensitive or not. However, on balance, I do not hold the authors to provide this extra evidence.
We agree that the proposed experiments would be interesting to perform for future studies and thank the reviewer for his comment.

We hope that the manuscript is now ready for publication.

REFERENCES
Johnson CH, Miwa I, Kondo T, Hastings JW (1989) Circadian rhythm of photoaccumulation in Paramecium bursaria. J Biol Rhythms. 4(4):405-415. 
Hasegawa K, Tanakadate A.J (1987) The Paramecium circadian behavioral rhythm: light phase response curves and entrainment. J. Biol Rhythms.  2(4):269-78.   
Morbiato E, Frigato E, Dinarello A, Maradonna F, Facchinello N, Argenton F, Carnevali O, Dalla Valle L, Bertolucci C (2019) Feeding Entrainment of the Zebrafish Circadian Clock Is Regulated by the Glucocorticoid Receptor. Cells. 8(11): 1342.
