# Supplementary material for: Contribution of the eye and of opn4xa function to circadian photoentrainment in the diurnal zebrafish
Source: PLoS Genet. 2024 Feb 26;20(2):e1011172. doi: 10.1371/journal.pgen.1011172 (PMC10919856; doi:10.1371/journal.pgen.1011172)
Supplement: Attachment — Submitted filename: answer to reviewers.docx [file pgen.1011172.s028.docx]

Dear editors and reviewers,

We thank the editors for their willingness to publish our work and are submitting a revised version of our manuscript. Please find a point-by-point answer to reviewer 2, as requested.

*1. Regarding the Δphase, I think that the difference between the final phase (‘phase after’) and the initial phase (‘phase before’) of the same larvae doesn’t faithfully reflect the effects of light impulses on phase changes of locomotor activities, even it might get a better value, as the authors argued. As shown in Figures 2D and 2E, compared to those in DD, light impulses at CT16 lead to phase delay, while light impulses at CT 21 cause phase advance. Thus Δphase between those of PD larvae and those of DD control larvae, and between those of PA larvae and those of DD control larvae should be a better indicator of the effects of light impulses on phase changes. With any behavioral assay, interindividual and interexperimental variations often occur, which should not be a reason not to calculate the right parameter. If the authors have calculated Δphase between those of PD larvae and those of DD control larvae, and between those of PA larvae and those of DD control larvae, they should describe them in the main text. I think that Δphase between the final phase (‘phase after’) and the initial phase (‘phase before’) of the same larvae is just arrow phase change, which will confuse the audience, should be deleted or put in to the supplementary file.*

We agree with the reviewer that calculating the difference between the DD and pulsed larvae before or after the pulse would have been interesting. However, we observed differences in the phase of DD versus pulsed larvae before any pulse application, as well as between experiments of the same type and therefore we do not feel that we would be able to interpret any differences observed after pulse application with this method. Instead, we used the standardization method described in the paper; this method was validated by the CBI statistician (M. Aguirrebengoa, personal communication).

*2. The authors said that “we addressed whether per2 and cry1a were induced upon administration of a one-hour light pulse at CT21 and how these inductions were affected by the absence of a functional lak gene. These results are described in figure 5”, I did not see them in Figure 5.*

This is a mistake on our part, the described results are in figure 3 and not 5. We apologize for this.

*3. The authors cited studies that paramecia display circadian rhythms, and argued that paramecia feeding could affect the larval locomotor rhythms. The fact is that the authors showed the amplitude damped quickly in their behavioral assays without feeding, while many behavioral experiments*

We are not aware of any studies having established that the type of feeding described by the reviewer does not interfere with entrainment of circadian rhythms by light nor of any other studies looking at circadian rhythms’ entrainment by light in zebrafish larvae while feeding them live preys. On the other hand, time-restricted feeding has a clear entraining effect (Morbiato et al. 2019), and paramecia have clear circadian rhythms which means that using them would result in time-restricted feeding, which is why we do not use it.

*4. I suggest the authors should cite the results of Biodare2, for example, locomotor activities of the
lak mutant and wild type display rhythmicity with statistically significant P values. Put the P
values on the respective panels. The author didnot give reasons why not putting the P
values on the respective panels.*

The method we use is to calculate the percentage of rhythmic larvae based on the ability of the Biodare2 algorithm to extract a period that is within the circadian range; it gives us a percent of rhythmic larvae rather than a P value. The advantage of this approach is that

1. It provides an evaluation of the rhythmicity within the circadian range (compared to other approaches which would provide an evaluation for rhythmicity in general, including with a periodicity outside of the circadian range)
2. The percentage of rhythmic larvae is easier to read than a p value assessing rhythmicity of each population. Indeed, these percentages allow for a straight forward comparison between control and mutant populations using a chi-two test

As previously mentioned, we have added the analysis we performed as supplemental tables and introduce it in the results section. We do not have with this method a p value to add to the panels.

*5. In Figure 5, tefalpha is still not changed into tefa.*

The change has been made.

We now hope the editors agree that our manuscript is ready for publication.

On behalf of the co-authors, sincerely

Elise CAU

**REFERENCES :**

Morbiato E, Frigato E, Dinarello A, Maradonna F, Facchinello N, Argenton F, Carnevali O, Dalla Valle L, Bertolucci C (2019) Feeding Entrainment of the Zebrafish Circadian Clock Is Regulated by the Glucocorticoid Receptor. Cells. 8(11): 1342.
